# Therapeutic effects of low-temperature plasma radiofrequency ablation and partial laryngectomy for glottis cancer: A comparative study

**DOI:** 10.12669/pjms.39.2.6847

**Published:** 2023

**Authors:** Kaiquan Zhu, Renyu Lin

**Affiliations:** 1 Kaiquan Zhu, Department of Otorhinolaryngology,The First Affiliated Hospital of Wenzhou Medical University, Wenzhou, Zhejiang Province, 325000 P.R. China; 2 Renyu Lin, Department of Otorhinolaryngology,The First Affiliated Hospital of Wenzhou Medical University, Wenzhou, Zhejiang Province, 325000 P.R. China

**Keywords:** Glottis laryngeal carcinoma, Low-temperature plasma radiofrequency ablation, Partial laryngectomy, Surgical treatment

## Abstract

**Objective:**

To compare the therapeutic effects of low-temperature plasma radiofrequency ablation and partial laryngectomy in the treatment of early glottis carcinoma.

**Methods:**

Clinical data of 80 patients with early glottis carcinoma treated in our hospital from June 2019 to January 2021 were analyzed. Patients were retrospectively divided into two groups based on the type of intervention. Forty patients received partial laryngectomy (Control group) and 40 patients received low-temperature plasma radiofrequency ablation (Observation group). Surgical indexes, length of hospital stay, postoperative complications, and visual analog scale (VAS) score of postoperative pain of patients in the two groups were compared. Postoperative stress response indexes, clinical efficacy, and postoperative recovery in two groups were compared and analyzed.

**Result:**

The operation time, hospital stay, intraoperative bleeding, and the incidence of postoperative complications in the observation group were significantly lower than those in the Control group (*P*<0.05). The postoperative pain VAS scores, Levels of malondialdehyde (MDA) and glutathione (GSH) in the observation group were significantly lower than those in the control group (*P*<0.05), while the level of nitro tyrosine (3-NT) and superoxide dismutase (SOD) were significantly higher than that in the control group (*P*<0.05). After a one-year follow-up, the excellent and good rate of pronunciation function in the observation group (95%) was significantly higher than control group (75%) (*P*<0.05).

**Conclusions:**

Low-temperature plasma radiofrequency ablation in the treatment of early glottis carcinoma is associated with less trauma, short operation time, less bleeding, short hospital stay and low postoperative stress reaction rate. Compared with partial laryngectomy, it has higher safety and better postoperative vocal cord function recovery.

## INTRODUCTION

Glottis carcinoma is a common type of carcinoma. It arises from the vocal cords and shows typical symptoms such as hoarseness and weak pronunciation at the very early stage of the disease, which makes it easy to diagnose.^1^ At present, the etiology of the disease is not completely clear. Studies suggest that it may be caused by a combination of environmental and occupational factors, living habits, viral infection, smoking, etc.^2^ Early glottis carcinoma is mainly treated by oral minimally invasive surgery, such as partial laryngectomy, which can effectively preserve the residual throat. However, this procedure is associated with long postoperative recovery time and changes in laryngeal function to a variable extent.^3^ Numerous studies indicate the benefits of using low-temperature plasma radiofrequency ablation technology in the field of head, neck, ear, nose and throat, since it is associated with less trauma and bleeding and fast postoperative recovery. In recent years, low-temperature plasma radiofrequency ablation was used as laryngeal minimally invasive surgery to treat early glottis carcinoma.^4^ In this paper, we compare the efficacy, pain level and safety of low-temperature plasma radiofrequency ablation and partial laryngectomy in the treatment of early glottis carcinoma. The purpose of this study was to provide a reference for clinical proper selection of surgical methods.

## METHODS

The records of 80 patients with early glottis carcinoma treated in our hospital from June 2019 to January 2021 were selected, including 61 males and 19 females. The average age of patients was 60.41±8.59 years. Patients were retrospectively divided into two groups based on the type of surgery. Forty patients that received partial laryngectomy comprised the control group, and 40 patients that were treated by low-temperature plasma radiofrequency ablation comprised the observation group.

### Inclusion criteria:

- Early glottis laryngeal carcinoma was diagnosed by pathological examination before the operation, and no lymph node metastasis was confirmed by neck CT.^5^
- All patients were treated for the first time.
- Age ≤ 75 years.
- Complete clinical data available.

### Exclusion criteria:

- Patients with severe dysfunction of the heart, liver, and kidneys.
- Severe diabetes, hypertension, and other primary diseases.
- Combined with other malignant tumors.
- Patients with exposure difficulties such as cervical lesions and deformities.

### Ethical approval:

This study was approved by the ethics committee of our hospital (Ref.: WZYY2022023; Date: January 18, 2022).

### Low-temperature plasma radiofrequency ablation:

After pre-operative preparation, a laryngoscopy examination was performed under general anaesthesia to confirm the location and size of the glottis lesion.

EIC7070-01 low-temperature plasma multifunctional surgical system (Smith & Nephew, UK) was used for surgery. The ablation power was set to seven and the hemostasis power was set to three. The tumor site was lifted with surgical forceps, and the tissue completely ablated 5mm at the edge of the tumor by whole block and multi-point resection. In patients with Tis, the acoustic ligament was preserved during the operation. In patients with T1, the affected side of the vocal cord was completely removed and ablated outward. From the laryngeal chamber to the exposed thyroid cartilage plate, 1/4 of the contralateral vocal cord was ablated forward. The anterior joint bone was fully exposed, and ablation performed downward to the upper edge of the trachea, and backward more than 5mm beyond the vocal cord process.

Hemostasis was secured using electrocoagulation. Finally, samples were taken at the peripheral and deep cutting edges respectively, and rapid frozen sections were taken for pathological examination. If the result were positive, it was necessary to continue to expand ablation and repeat pathological examination of the frozen sections until the pathological result of the cutting edge were negative.

### Partial laryngectomy:

After pre-operative preparation, a T-shaped incision in the middle of the neck was given, tissue was gradually separated, fully exposing the laryngopharyngeal cavity and defining the cervical lymph nodes. While monitoring for active bleeding, the anterior laryngeal banded muscle was separated from the white line, exposing the laryngeal body. The incision was made to cut into the laryngeal cavity from the cricothyroid membrane, the thyroid cartilage along the tumor side of the white line was bit with bone biting forceps, the banded muscle fascia was separated for standby, expanded 0.5 cm from the edge of the tumor.^6^

Tumor was completely removed, and the wound was repaired, bleeding stopped, the incision sutured, and the operation completed. Cephalosporin antibiotics were given 30 minutes before the operation antibiotic treatment was continued three to five days after the operation, and budesonide and normal saline were atomized for seven days. Postoperative vocal cord rest (silence) for two weeks, and regular reexamination after operation were performed for all the patients.

The following basic clinical data and relevant indexes were collected. Operation time, intraoperative bleeding, postoperative complications, length of hospital stay, and pain score (one, three, and seven days after the operation, VAS was used to evaluate the pain score, with a total of 10 points. The lower the score, the lighter the pain).^7^

Twenty-four hours after the operation, the levels of stress response indexes such as malondialdehyde (MDA), nitro tyrosine (3-NT), superoxide dismutase (SOD), and glutathione (GSH) in peripheral venous blood were measured by enzyme-linked immunosorbent assay (ELISA) (the reagent was purchased from Beijing Jingmei Bioengineering Co., Ltd.).

Postoperative treatment effect efficacy evaluation was labelled complete remission (CR) when the symptoms, signs, and lesions disappeared completely, the pronunciation function and swallowing function returned to normal, and there was no recurrence or metastasis within one year; Partial remission (PR) was declared when the symptoms and signs were significantly improved, the tumor focus area was reduced by more than 75%, the maintenance time was more than 12 months, there was no metastasis, and the pronunciation and swallowing function did not affect daily life .If the pronunciation and swallowing function were significantly improved, the product of two diameters of tumor lesions was reduced by 50% ~ 75% and there were no new lesions, the condition was defined as stable (SD). New lesions, metastases and aggravation were considered progression (PD).^8^ Total effective rate = (CR + PR) / total number of patients×100%.

The recovery effect one year after operation, including pronunciation function, swallowing function, recurrence, survival and so on. Pronunciation function: Excellent - clear and coherent voice, normal communication; Good - clear and coherent voice with mild hoarseness; General - poor clarity and continuity of voice, which has a certain impact on life and work; Poor - The voice is not clear and cannot communicate normally. To assess swallowing function: X-ray fluoroscopy swallowing examination was adopted, with a total score of 10 points, including severe abnormality (0-2 points), moderate abnormality (3-6 points), mild abnormality (7-9 points) and completely normal (10 points).^9^

### Statistical analysis:

For the sample size calculation, we assumed that the survival rate of low-temperature plasma radiofrequency ablation (90~95%) and partial laryngectomy (60%~70%) in the treatment of early glottis carcinoma according to the previous studies.^10^-^12^ A power of 80%, significance level of 95% and a possibility of incomplete records of 10% were set, and it is calculated that a minimum sample size of 34 was required for the study.^13^ The data of this study were processed by SPSS 22.0 professional statistical software. The measurement data were expressed by () and t-test was carried out; the counting data were expressed by “n (%)” and *χ^2^* test was performed. P<0.05 was considered statistically significant.

## RESULTS

A total of 80 patients met the inclusion criteria. The control group consisted of 40 patients, including 29 males and 11 females, with an average age of 59.57±8.72, the clinical stages were: 17 patients in the Tis stage, 10 patients in the T1a stage, 10 patients in the T1b stage, and three patients in T2N0M0 stage; There were 40 patients in the observation group, including 32 males and eight females, with an average age of 61.25±8.47, clinical staging: 17 patients in Tis stage, nine patients in T1a stage, eight patients in T1b stage, and six patients in T2N0M0 stage;. There was no significant difference in sex, average age, and clinical stage between the two groups (*P*>0.05).

The operation time, hospital stay, intraoperative bleeding, postoperative complications, and pain VAS score of the patients in the observation group were significantly lower than those of the control group (*P*<0.05) Table-I.

**Table-I:** Comparison of surgical indexes between the two groups (N=80).

Group (n)	Operation time (minute)	Intraoperative bleeding (ml)	Hospital stay (day)	Incidence of complications n(%)	Pain VAS score		

					One day after operation	Three day after operation	Seven day after operation
Control group	49.85±7.92	83.77±8.16	11.15±1.95	10 (25.0)	7.02±1.25	5.22±0.89	2.15±0.48
Observation group	15.15±2.72	13.57±2.35	5.12±1.43	3 (7.5%)	4.35±1.00	2.50±0.55	0.92±0.47
*t*/*χ^2^*	26.201	52.261	15.709	4.501	10.561	16.418	11.444
*P*	<0.001	<0.001	<0.001	0.034	<0.001	<0.001	<0.001

Twenty four hours after the operation, the levels of MDA and GSH in the observation group were significantly lower, and the levels of SOD and 3-NT were significantly higher than those in the control group (*P*<0.05); There was no significant difference in the total efficacy between the observation group and the control group (*P*>0.05) Table-II.

**Table-II:** Comparison of 24-hour stress response indexes and total effective between the two groups (*χ̅*±*S*). (N=80)

Group	n	MDA/(mmol/L)	3-NT/(ng/L)	SOD/(IU/mL)	GSH/(IU/mL)	Total effective				

						CR	PR	SD	PD	Total effective rate
Control group	40	11.58±1.67	58.22±7.82	63.20±5.84	1.25±0.32	18 (45.00)	19 (47.50)	2 (5.00)	1 (2.50)	37 (92.50)
Observation group	40	8.25±1.02	71.25±7.66	81.35±8.13	0.75±0.11	20 (50.00)	18 (45.00)	1 (2.50)	1 (2.50)	38 (95.00)
*χ^2^*/t		10.785	-7.521	-11.462	9.131	0.213				
*P*		<0.001	<0.001	<0.001	<0.001	0.644				

One year after the operation, the rate of excellent and good pronunciation function in the observation group was significantly higher than that in the Control group (P<0.05), but there was no significant difference in swallowing function, recurrence rate and survival rate between the two groups (*P*>0.05) Table-III.

**Table-III:** Comparison of postoperative recovery effects between the two groups (n%). (N=80)

Group (n)	Pronunciation function				Swallowing function				Recurrence rate	1-year survival rate

	Excellent	Good	Average	Poor	Normal	Light	Moderate	Severe		
Control group	10(25.0)	20(50.0)	9(22.5)	1(2.5)	15(37.5)	19(47.5)	6(15.0)	0(0)	2(5.0)	39(97.5)
Observation group	20(50.0)	18(45.0)	2(5.0)	0(0)	17(42.5)	18(45.0)	5(12.5)	0(0)	0(0)	40(10)
*χ^2^*	8.893				0.243				0.346	1.013
*P*	0.031				0.886				0.556	0.314

## DISCUSSION

The results of this study suggest that there is no significant difference between the total efficacy of 95.00% in the Observation group and 92.50% in the control group, indicating that low-temperature plasma radiofrequency ablation has achieved significant therapeutic effect, similar to that of the conventional partial laryngectomy. Compared with partial laryngectomy, low-temperature plasma radiofrequency ablation is associated with shorter operation time, less intraoperative bleeding, reduced length of hospital stays and lower incidence of complications. Patients treated with low-temperature plasma radiofrequency ablation also report lower pain scores, which makes this procedure more conducive to the rehabilitation of patients. The research of Zhang Y *et al*^10^ also showed that low-temperature plasma radiofrequency ablation resulted in shorter operation time, faster recovery of vocal cord structure, vibration and speech function.

The low-temperature plasma multifunctional surgical system has the functions of cutting, ablation, hemostasis and flushing, and the knife head is flexible and easy to operate. With the assistance of the imaging system, there is no need to cut the trachea, which reduces the pain, and effectively improves the accuracy and safety of the procedure.^14^ Similarly, another study revealed that plasma low-temperature radiofrequency ablation to treat early glottis carcinoma reduced the operation time which was no more than 20min, the amount of bleeding was no more than 15ml, and the hospital stay did not exceed one week.^15^,^16^

Surgical trauma activates systemic oxidative stress response and produce too many oxygen free radicals that may cause tissue and cell damage.^17^ SOD and GSH are the main antioxidant enzymes, which can specifically scavenge harmful free radicals, prevent the damage caused by free radical oxidation and keep the local environment in a reduced state.^18^ However, surgical trauma may lead to over production of oxygen free radicals that will exceed the scavenging capacity of SOD and GSH. This in turn may cause peroxidation and produce a variety of peroxidation products in the cells. For example, MDA and 3-NT are common peroxidation products in vivo, which can reflect the degree of oxidative stress.^19^ The results of our study showed that the levels of MDA and GSH in the Observation group 24 hours after the operation were significantly lower than those in the control group, and the levels of SOD and 3-NT were significantly higher than those in the control group, indicating that low-temperature plasma radiofrequency ablation is more efficient in reducing surgical stress response of the patients.

The recovery of postoperative pronunciation and swallowing function is one of the main concerns in carcinoma surgery. Studies have showed that the treatment of glottis carcinoma with traditional open laryngectomy is associated with extensive damage to the laryngeal tissue that affects postoperative pronunciation and swallowing functions. In serious cases, even the respiratory function may be significantly affected, which greatly reduces the postoperative quality of life.^20^,^21^ After a one-year follow-up, the recovery of pronunciation function in the Observation group was significantly better than that in the control group.

Plasma low-temperature radiofrequency ablation technology achieves the effect of cutting and ablation of diseased tissues at low temperature (40~70°C), which effectively reduces the damage to the surrounding tissues and facilitates fast recovery of postoperative pronunciation, swallowing and respiratory functions. However, there was no significant difference in swallowing function between the two groups, which may be due to the small sample size and short follow-up time of this study. Nachalon Y et al.^22^ study found that one month after low-temperature plasma radiofrequency ablation, the pronunciation function of patients with early glottis carcinoma was significantly improved. Three months after the operation, the pronunciation basically returned to normal. Puram SV et al.^23^ showed that in the treatment of patients with early glottis carcinoma low-temperature plasma radiofrequency ablation eliminates the need to cut the throat and completes the ablation of the focus at low temperature. This allows to effectively retain the morphology and physiological structure of the throat, and contributes to faster recovery of swallowing and pronunciation functions after the operation.

The results of this study suggest that the curative effect of low-temperature plasma radiofrequency ablation is equivalent to that of partial laryngectomy, it can completely eliminate the tumor focus, and is associated with significantly lower rate of postoperative complications compared to standard surgical procedure. Since partial laryngectomy requires the incision, it may lead to the direct damage to the integrity of laryngeal respiratory tract mucosa, laryngeal sphincter and nerve.

Moreover, open throat surgery increases the risk of respiratory tract infection, affects swallowing function, increases the risk of aspiration pneumonia. The resulting surgical wound is large, and may easily cause laryngeal stenosis and granulation tissue hyperplasia.^24^ Elicin O et al.^25^ and others believe that low-temperature plasma radiofrequency ablation technology can not only effectively avoid the shortcomings of partial laryngectomy, but also improve the accuracy and safety of the surgery, and accelerate the rehabilitation of patients.

### Limitation of the study:

Due to the insufficient sample size and short follow-up time, the accuracy of the results needs to be confirmed by more clinical studies. Therefore, in the future, it is necessary to expand the sample size and continue in-depth research to further observe the long-term effect.

## CONCLUSION

In the treatment of early glottis carcinoma, low-temperature plasma radiofrequency ablation has the advantages of clear vision, flexible knife head, simple procedure and high accuracy. Compared with partial laryngectomy, it is associated with shorter operation time, less bleeding, fewer complications, less pain and faster postoperative recovery.

### Authors’ contributions:

**KZ** conceived and designed the study.

**KZ and RL** collected the data and performed the analysis.

**KZ** was involved in the writing of the manuscript and is responsible for the integrity of the study.

All authors have read and approved the final manuscript.
